# Immunotherapeutic strategies in head and neck cancer: challenges and opportunities

**DOI:** 10.1172/JCI188128

**Published:** 2025-04-15

**Authors:** Xia Liu, R. Alex Harbison, Mark A. Varvares, Sidharth V. Puram, Guangyong Peng

**Affiliations:** 1 Department of Otolaryngology–Head and Neck Surgery,; 2 Rob Ebert and Greg Stubblefield Head and Neck Tumor Center at Siteman Cancer Center and; 3 Department of Pathology & Immunology, Washington University School of Medicine, St. Louis, Missouri, USA.; 4 Department of Otolaryngology–Head and Neck Surgery, Massachusetts Eye and Ear, Harvard Medical School, Boston, Massachusetts, USA.; 5 Department of Genetics, Washington University in St. Louis, St. Louis, Missouri, USA.

## Abstract

HNSCC remains a substantial health issue, with treatment options including surgery, radiation, and platinum-based chemotherapy. Unfortunately, despite progress in research, only modest gains have been made in disease control, with existing treatments resulting in significant functional and quality-of-life issues. The introduction of immunotherapy in the treatment of HNSCC has resulted in some improvements in outlook for patients and is now standard of care for populations with both recurrent and metastatic disease. However, despite the early successes, responses to immune checkpoint inhibition (ICI) remain modest to low, approaching 14%–22% objective response rates. Challenges to the effectiveness of ICI and other immunotherapies are complex, including the diverse and dynamic molecular plasticity and heterogeneity of HNSCCs; lack of immunogenic antigens; accumulated suppressive immune populations such as myeloid cells and dysfunctional T cells; nutrient depletion; and metabolic dysregulation in the HNSCC tumor microenvironment. In this Review, we explore the mechanisms responsible for immunotherapy resistance, dissect these challenges, and discuss potential opportunities for overcoming hurdles to the development of successful immunotherapy for HNSCC.

## Introduction

Head and neck squamous cell carcinoma (HNSCC) is the sixth most common cancer globally, with approximately 800,000 new cases and approximately 450,000 deaths annually (1). Treatment of HNSCC has made modest advances over the course of decades, consisting of cytotoxic platinum-based chemoradiation (CRT) or primary surgical management with risk-adapted adjuvant therapy (i.e., radiation and/or chemotherapy). With current treatment strategies, more than 50% of patients experience recurrence within 3 years, while more than 10% experience distant failures (2–4). Moreover, surgery, radiation, and chemotherapy all harbor significant side effects. Unfortunately, HNSCC lacks actionable genomic targets due to the complex and evolving genomic landscape, with limited success in targeting EGFR and PIK3CA (5, 6). Therefore, understanding the molecular mechanisms responsible for HNSCC pathogenesis and development of effective therapeutic strategies are substantial hurdles in the management of HNSCC.

The emergence of immune checkpoint inhibition (ICI) represents a major shift in the treatment paradigm of primary and recurrent/metastatic (R/M) HNSCC. ICI is now the standard of care (SoC) for R/M HNSCC (7), but response rates remain modest, around 14%–22% (8, 9). Other immunotherapeutic strategies, including tumor vaccines and adoptive transfer of antigen-specific T cells, have also been explored in clinical trials involving patients with HNSCC with limited response rates (10, 11). Several potential factors contribute to these modest responses, including tumor-intrinsic molecular heterogeneity and metabolic adaptations, which diminish antitumor immunity, tumor antigen escape, influx of suppressive immune cell populations, dysregulated metabolism, and development of dysfunctional antitumor T cells (12–16). To overcome these formidable obstacles and advance the outlook for patients, better understanding of the mechanisms driving resistance to antitumor immunity is urgently needed.

In this Review, we provide an update on the current landscape of immunotherapy in patients with HNSCC, explore potential mechanisms driving resistance to immunotherapy, discuss major challenges in the immunotherapy of HNSCC patients, and offer our perspective on prioritizing development of targets influencing immunotherapy outcomes.

## Current immunotherapeutic strategies in HNSCC

While current immunotherapeutic strategies for HNSCC primarily leverage ICIs, such as α–PD-1 therapies, a variety of approaches including T cell–based immunotherapy are emerging. The advent of ICIs, particularly PD-1 inhibitors such as nivolumab and pembrolizumab, has transformed the treatment landscape for HNSCC. While the impact of ICIs in HNSCC has not matched that in melanoma (17) or cutaneous squamous cell carcinoma (18), the landmark trials CheckMate 141 and KEYNOTE-040 galvanized the use of ICIs in the treatment of R/M HNSCC (8, 9). Moreover, forthcoming data from the KEYNOTE-689 trial will be paradigm-shifting (19). Critical questions remain at the forefront of ongoing research: What is the optimal timing for immunotherapy administration — adjuvant or/and neoadjuvant? sequential or concurrent? How can predictive biomarkers refine patient selection? And how can immunotherapies best integrate with established SoC? These challenges reflect the rapidly evolving landscape of HNSCC immunotherapy. An overview of current evidence and future directions of immunotherapy is provided in Tables 1–5, setting the stage for the next chapter in this transformative field.

### Combination ICI.

Initial ICI trials in HNSCC demonstrated durable treatment responses and overall survival (OS), suggesting maintenance of immune equilibrium (Table 1). Combination ICI with multiple coinhibitory molecules may amplify the treatment response by differentially regulating various cell populations in the tumor microenvironment (TME) (20). The CheckMate 651 study, combining α–PD-1 and α–CTLA-4 blockade in R/M HNSCC, observed no change in objective response rate (ORR) or OS (21). Similarly, CheckMate 714 observed no change in ORR with α–PD-1/α–CTLA-4 inhibition over α–PD-1-alone in platinum-refractory R/M HNSCC (22). The phase III EAGLE trial evaluated durvalumab, an α–PD-L1 monoclonal antibody, versus durvalumab plus tremelimumab (α–CTLA-4) versus SoC in patients with R/M HNSCC. OS did not differ across groups relative to SoC (23). In parallel, KESTREL found no benefit to single-agent or combination α–PD-L1 with or without α–CTLA-4, even noting that patients with high PD-L1 expression receiving SoC had better ORR compared with durvalumab alone or durvalumab plus tremelimumab (24). While reinvigoration of cytotoxic T cell function through coregulatory signal pathway modulation is effective, deeper understanding of the mechanisms regulating the fate and response of effector lymphocytes in TME is critically needed.

### Radiation therapy in combination with ICI.

Ionizing radiation is under active investigation for enhancing immunotherapeutic responses. Putative mechanisms supporting this approach are the activation of cytotoxic lymphocytes, DC activation and T cell priming, activation of pro-death signaling in tumor cells, and release of damage-associated molecular patterns (DAMPs) (25). A phase I study testing the safety of partial tumor irradiation with stereotactic body radiotherapy to oligometastatic disease coupled with pembrolizumab in advanced solid tumors demonstrated encouraging results (Table 2) (26). In contrast, NRG-HN004 (ClinicalTrials.gov NCT03258554) compared radiotherapy with concurrent plus adjuvant durvalumab versus RT/cetuximab, observing no improvement in progression-free survival (PFS) (27). However, irradiation also induces deleterious effects, including increased Treg and myeloid-derived suppressor cell (MDSC) infiltration, PD-L1 induction, and activation of prosurvival mechanisms via chronic IFN signaling (25, 28). The role of radiotherapy in modulating antitumor immunity remains to be elucidated as a tool for enhancing immunotherapy effectiveness.

### Chemoradiotherapy combined with ICI.

Building on the advances of KEYNOTE-040 and CheckMate 141 in R/M HNSCC, exciting advances are on the horizon integrating immunotherapy with definitive CRT (Table 2). The role of SoC cisplatin chemotherapy for enhancing ICI is under active investigation, with support from preclinical data (29, 30). Preclinical and clinical data suggest that cisplatin promotes immunogenic tumor cell death (30), DC activation, and antigen-specific T cell killing (31). Data evaluating concurrent versus sequential pembrolizumab in the definitive treatment of HNSCC with CRT highlight better outcomes with sequential immunotherapy (32). The phase III KEYNOTE-048 trial comparing pembrolizumab with and without chemotherapy with cetuximab plus chemotherapy for R/M HNSCC revealed a lack of PFS benefit with ICI (33). Pembrolizumab with chemotherapy improved OS compared with cetuximab plus chemotherapy in all subpopulations, independent of PD-L1 combined positive score (CPS) status. The phase III KEYNOTE-412 (NCT03040999) trial evaluated pembrolizumab plus CRT versus CRT in locally advanced HNSCC (LA-HNSCC), finding no difference in event-free survival (34). The JAVELIN trial comparing avelumab plus CRT versus CRT in LA-HNSCC also observed no difference in PFS (35). Further work is warranted to elucidate the extent to which chemotherapy can enhance ICI effectiveness.

### RTK inhibition and ICI.

Combining ICI with RTK inhibition is promising, though recent trial results were modest (Table 3). EGFR is an established therapeutic target in HNSCC. There is a propensity for *EGFR* copy number amplification and overexpression in carcinogen-driven, HPV^–^ HNSCCs. Cetuximab, an EGFR-targeting mAb, was one of the first immunotherapies approved for HNSCC, marking a pivotal moment in systemic cancer therapy. Cetuximab can augment antitumor immunity, promoting DC maturation, CD8^+^ T cell priming, NK cell functions, and antibody-dependent cell-mediated cytotoxicity (ADCC) (36, 37). Compared with SoC cisplatin, cetuximab offers unique benefits, particularly for patients unable to tolerate cisplatin, though its utility has been limited by the side effect of acneiform rash (38). Initial studies demonstrated the advantages of combining cetuximab with radiotherapy, showing improved outcomes compared with radiotherapy alone (39). Promising results were also observed with pembrolizumab and cetuximab in combination in a phase II trial among patients with platinum-ineligible or -resistant R/M HNSCC (40). However, GORTEC-REACH — comparing concurrent cisplatin or cetuximab radiotherapy versus radiotherapy with concurrent weekly cetuximab and avelumab (α–PD-L1) — failed to meet its primary end point (41). Despite these limitations, cetuximab facilitated broader adoption of immunotherapies in HNSCC and bridged the gap to the next generation of immunotherapies, leaving a lasting legacy in HNSCC treatment.

In addition to cetuximab, VEGF inhibitors, including tyrosine kinase inhibitors (TKIs), have immunomodulatory properties. A phase II trial combining pembrolizumab and cabozantinib, a multikinase TKI, observed a partial response or stable disease in over half the cohort in conjunction with increased CD8^+^ T cell infiltrates in responders (42). The ALPHA study combining pembrolizumab with afatinib, an irreversible TKI, observed a promising ORR (43). The KEYNOTE-146 phase IB/II trial of lenvatinib plus pembrolizumab found an encouraging response rate in the phase II expansion cohort (44). However, the LEAP-010 phase III study (NCT04199104) combining first-line pembrolizumab with or without lenvatinib was discontinued after OS failed to improve (45). Given the variability in these results, a biological approach to identify and overcome barriers to effective antitumor immunity in HNSCC is warranted.

### Antitumor vaccine therapy.

Vaccine-based immunotherapy for HPV^+^ HNSCC is a logical intervention to target tumor cells expressing viral antigens (e.g., E6/E7) (46). Several strategies — including live-vector vaccines (e.g., axalimogene filolisbac secreting the Lm-LLO-HPV E7 fusion protein), peptide vaccines such as ISA101 in combination with ICI (NCT03669718, NCT04398524, and NCT04369937), and the DNA vaccine MEDI0457 — have been developed and tested in patients with HPV^+^ cervical and oropharyngeal squamous cell carcinoma (OPSCC), with modest results (Table 4). One hurdle is overcoming T cell dysfunction in the TME with vaccine-mediated approaches. Several groups are examining combinations of ICI with anticancer vaccines, including a vaccinia virus encoding E6/E7 combined with IL-2 plus α–PD-L1 (NCT03260023) (47); a liposomal-based HPV16 E6/E7 peptide vaccine (PDS0101) in combination with pembrolizumab (NCT04260126 and NCT05232851) (48); and the SQZ-PBMC-HPV vaccine in combination with atezolizumab, ipilimumab, and nivolumab in patients with R/M HPV16^+^ solid tumors (NCT04084951). Additionally, a novel fusion protein in combination with pembrolizumab (HPV16 E7-pHLA-IL2-Fc) is under investigation at several centers (NCT03978689), with results suggesting expansion of E7_11–20_–specific clonotypes (49). Identifying high-affinity tumor antigens while avoiding cross-reactivity with host proteins and emergence of poorly immunogenic neoantigens remains a challenge in vaccine-based therapy in HNSCC.

### Adoptive T cell therapy.

Adoptive T cell therapy (ACT) — infusing tumor-reactive T cells, expanded tumor-infiltrating T cells (TILs), gene-engineered T cell receptor T (TCR-T) cells, and CAR T cells — represents an opportunity to leverage antigen specificity in HNSCC treatment, though there is a paucity of known antigens in HNSCC (Table 4). Previously, Hinrichs’s group tested ACT using TILs selected for HPV E6 and E7 reactivity (11). TIL therapy is limited by the lengthy process of isolating and expanding TILs, as well as the need for surgical tumor excision from patients. Alternatively, TCR-T cell manufacturing decreases production time. TCR-T cell therapy has been accomplished in HPV-related cancers including cervical cancer and OPSCC using autologous E7 TCR-T cells (50). In a phase I trial of HPV16 E7 TCR-T cell therapy, 50% of patients responded, including several with α–PD-1–refractory disease (50). However, limited progress has been made in the development of CAR T–based therapies in HNSCCs. Although ACT offers the advantage of specifically targeting tumor cells compared with the other immunotherapy strategies, it still faces formidable challenges in the suppressive TME, such as nutrient deprivation, suppressive metabolites, and regulatory immune cell interactions. Combining tumor-specific T cell therapies with agents that overcome these limitations in the TME should increase the effectiveness of this approach.

## Mechanisms diminishing the response to current immunotherapeutic strategies

**Figure 1:**
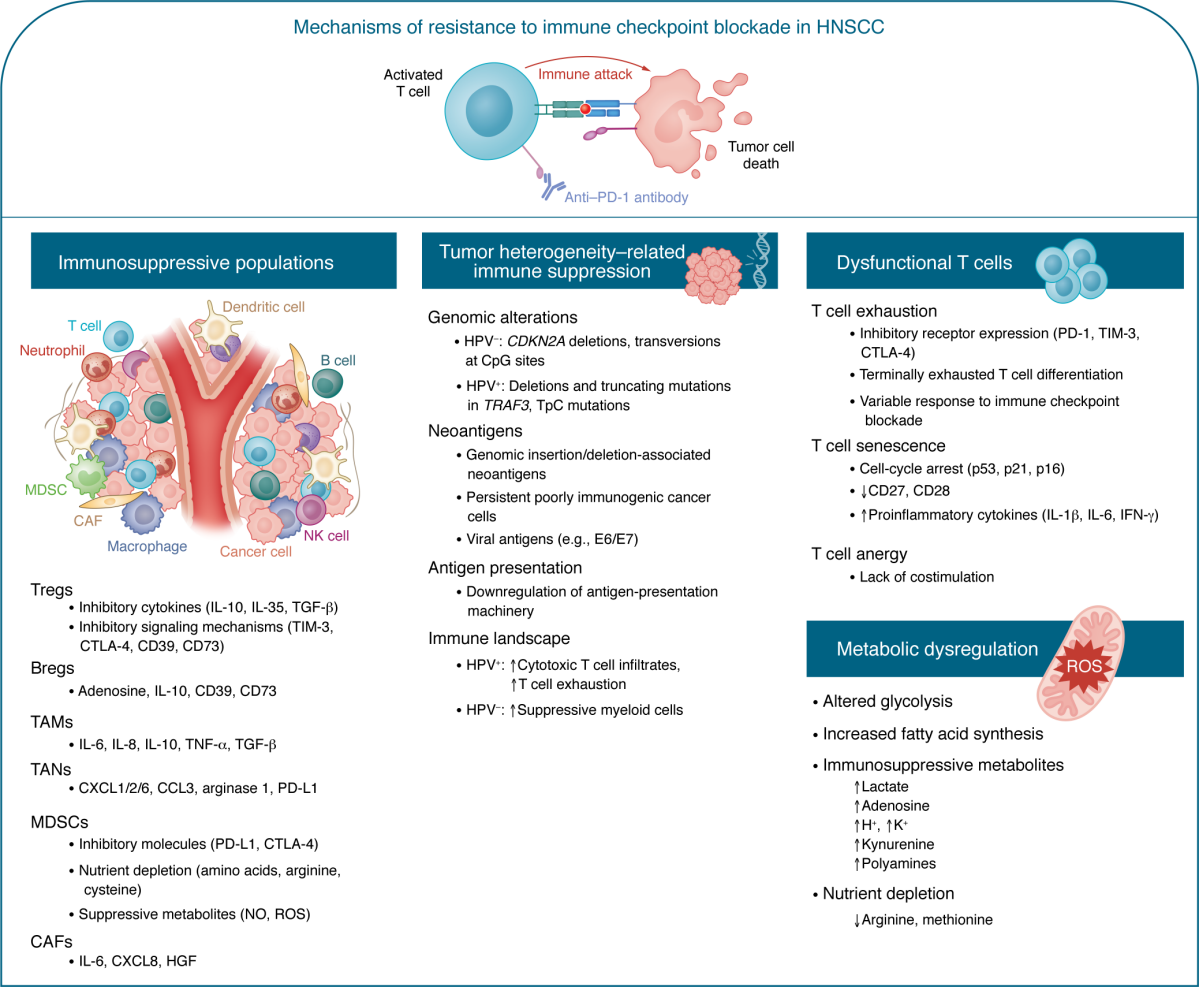
Mechanisms driving resistance to immune checkpoint blockade therapy in HNSCC. The potential mechanisms driving resistance to antitumor immune responses are illustrated. Overcoming immune-suppressive populations including Tregs, Bregs, tumor-associated macrophages (TAMs), tumor-associated neutrophils (TANs), myeloid-derived suppressor cells (MDSCs), and cancer-associated fibroblasts (CAFs), as well as immune plasticity, will be critical for enhancing the response to immunotherapy and antitumor immunity. Defining cell-intrinsic features of HPV-related and carcinogen-driven (e.g., smoking) HNSCC will also be fundamental for reducing their tumor-intrinsic immune-suppressive capacity and immune escape mechanisms. Dysfunctional T cells generated by chronic antigen stimulation or T cell senescence induced by the tumor metabolome, proteome, and chemokine/cytokine milieu also impair the effectiveness of the immune response in HNSCC. Inadequate T cell costimulation drives T cell anergy, further impairing this response. Less-well-understood mechanisms driven by metabolic dysregulation impact the antitumor immune response in the tumor microenvironment, for which further work is warranted.

Given the limited success with current immunotherapies in HNSCC, identification of the mechanisms responsible for immunotherapeutic resistance is urgently needed. Potential mechanisms are summarized below and shown in Figure 1.

### Molecular and immune heterogeneity of HNSCC.

**Figure 2:**
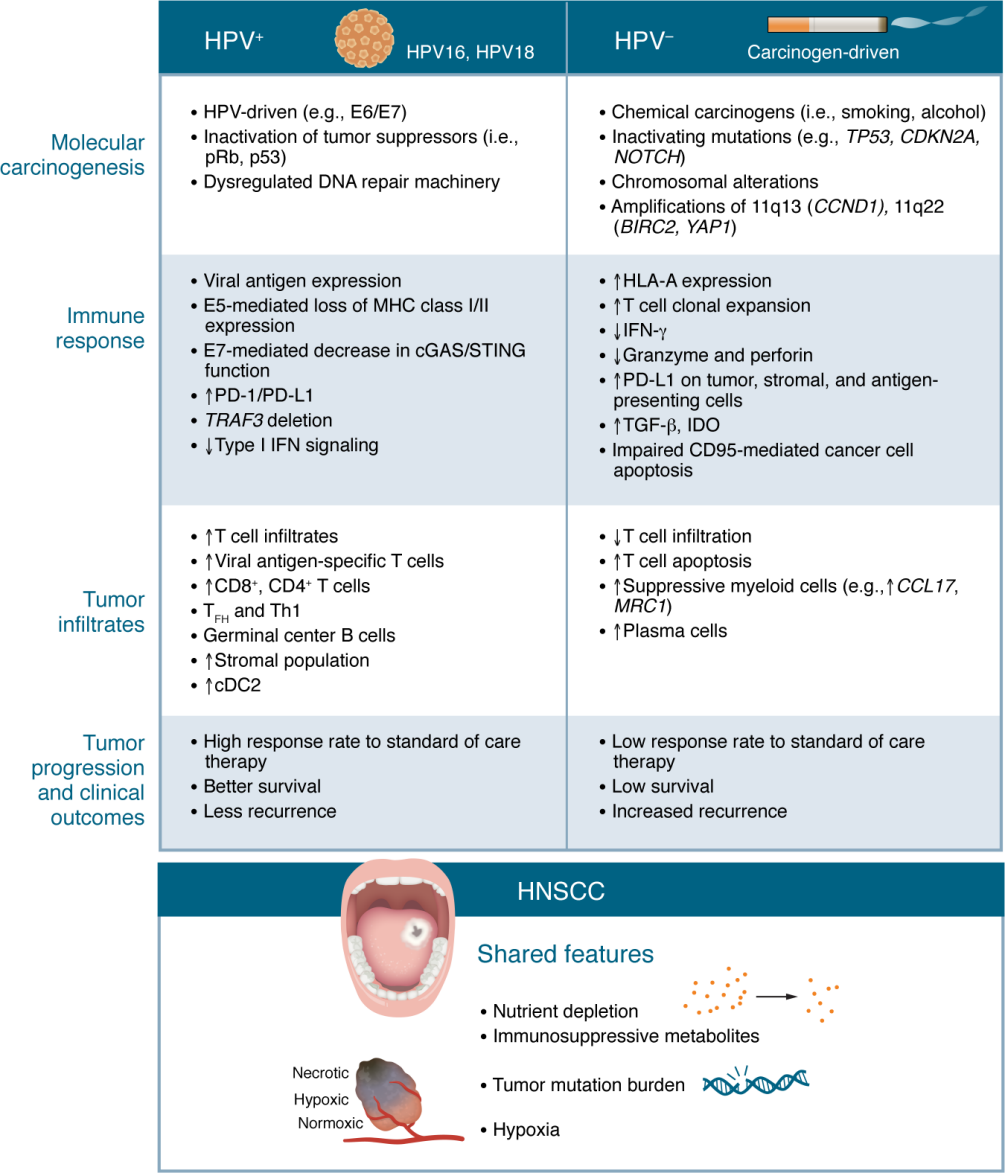
Features driving distinct immune pathogenesis in HPV^+^ versus HPV^–^ HNSCC. Unique etiologies of HNSCC drive differential immune responses and pathogenesis. HPV^+^ HNSCCs are driven by dominant viral antigens (e.g., E6/E7), altered immune checkpoint signaling, and diminished cytosolic DNA–sensing functionality resulting in high levels of T lymphocyte infiltrates and germinal center B cells. In comparison, carcinogen-driven HNSCCs harbor a myeloid-rich, immune-suppressive tumor microenvironment driven by release of regulatory signaling molecules, genomic heterogeneity, and a lack of highly immunogenic neoepitopes. In HPV^+^ HNSCCs, viral oncoproteins promote loss of tumor suppressor gene expression and viral protein–mediated impairment of immunogenicity and antigen presentation. In HPV^–^ HNSCCs, carcinogens such as those found in tobacco smoke impair CD8^+^ T cell function. Collectively, these differences are associated with generally better survival outcomes in HPV^+^ HNSCC compared with HPV^–^ HNSCC. cDC2, type 2 conventional DCs.

HNSCC tumorigenesis is driven by HPV (HPV^+^) and/or carcinogens (e.g., smoking and alcohol). Carcinogen-driven (i.e., HPV^–^) HNSCC is largely mediated by loss-of-function mutations in tumor-suppressor genes (e.g., *TP53* and *CDKN2A*), whereas HPV^+^ HNSCC carcinogenesis is driven by viral oncoprotein–mediated inactivation of tumor-suppressor genes (6). Tumor molecular heterogeneity drives different immune pathogeneses via several mechanisms (Figures 1 and 2). While mutation rates do not differ by HPV status, there are differences in where mutations tend to occur (e.g., CpG sites) (6). Genomic heterogeneity and instability potentially drive additional mechanisms promoting immune escape (51). Prevalent somatic mutations and indel-derived tumor-specific neoantigens partially account for this heterogeneity. Implications for antitumor immunity include the emergence of dominant tumor antigens that suppress the function of other TCR clonotypes (52). Moreover, cancer immunoediting results in persistent poorly immunogenic cancer cells that can escape the equilibrium phase (53, 54).

More nuanced differences between HPV^+^ and HPV^–^ HNSCCs also drive variable immune phenotypes and responses, as illustrated in Figure 2. HPV^+^ and HPV^–^ HNSCCs share some immune features, such as a metabolic milieu detrimental to antitumor immunity (55), chronic antigen stimulation, and variable Treg infiltrates (55–57), but also possess distinct features. HPV^–^ HNSCCs have a more immune-suppressed TME, with high frequencies of PD-1–expressing CD4^+^ Th1 cells, accumulation of tumor-associated macrophages (TAMs) and MDSCs, and high MHC expression and tumor immunogenicity of tumor antigens (56–61). HPV^+^ HNSCCs are unique in that the causal agent also accounts for immunogenic antigens provoking tumor-specific responses. HPV^+^ tumors are notable for enrichment of conventional CD4^+^ and CD8^+^ T cells, B cell subsets, and stromal cells and enriched HPV-specific T cells, as well as exhausted T cells (62–66) (Figure 2). Emerging data reveal that HPV gene expression is variable across HPV^+^ HNSCCs, which may represent another mechanism of immune evasion by these tumors (12). Further work is needed to define the unique molecular and biochemical features driving immune pathogenesis in HPV^+^ and HPV^–^ HNSCCs.

### Suppressive tumor-infiltrating immune cell populations.

Malignant tumors can recruit and/or develop different types of suppressive cells in the TME, such as Tregs (14), tumor-associated neutrophils (TANs) (67), MDSCs (15), TAMs (16), and cancer-associated fibroblasts (CAFs), which promote cancer progression and immune escape, and induce immunotherapy resistance in HNSCC (Figure 1).

### CAFs.

An abundance of CAFs is found in the stroma, constituting up to 80% of the cellular composition in late-stage HNSCC (68). CAFs play an important role in HNSCC tumor growth, facilitating proliferation, invasion, migration, and angiogenesis, and promoting treatment resistance (13). Several subtypes of CAFs accumulate in HNSCCs, with most exhibiting protumoral function, such as myofibroblasts with high α–smooth muscle actin (α-SMA) expression, extracellular matrix–expressing (ECM-expressing) CAFs, and MHC II^+^ CAFs (69, 70). Furthermore, exploration of another phenotype of CAFs expressing elastic fiber differentiation genes revealed a negative prognostic impact on HPV^+^ HNSCC (69, 70). CAFs can affect tumor cells and immune cells in the TME via multiple mechanisms: (i) The signaling regulatory loop of CAF-derived HGF and HNSCC-derived basic FGF (bFGF) increases oxidative phosphorylation (OXPHOS) in CAFs and glycolysis in HNSCC cells (68); (ii) CAFs and their supernatants suppress T cell proliferation and promote Treg functions (71); (iii) CAFs induce immunotherapy resistance via CD8^+^ T cell exclusion (72); (iv) CAFs secrete a number of factors that induce protumoral and immunosuppressive macrophage differentiation from monocytes, which suppresses T cell proliferation (73); and (v) CAF-derived TGF-β promotes cetuximab resistance in HNSCC preclinical models (74).

### TAMs.

TAMs are a major tumor-infiltrating immune cell subset in HNSCC, playing a key role in tumor growth (75, 76). M2 macrophage infiltrates correlate with aggressive tumor features, lymph node metastases, and poor prognosis in HNSCC (76–78). TAMs also correlate with aggressive clinicopathologic features in HNSCC (16). Under hypoxic stress, TAMs secrete TNF-α, IL-1, IL-6, IL-8, VEGF, GM-CSF, TGF-β, and MMP, promoting tumor angiogenesis and invasion (79). TAMs are the major source of PD-L1 and other immune checkpoint ligands in the HNSCC TME (69). PD-L1^+^ TAMs are closely associated with CD8^+^ T cell function, suggesting regulatory cell-cell interactions in HNSCC (69). In addition, TAMs express PD-1, which decreases their phagocytic and cytotoxic potency (80).

### MDSCs.

Infiltration of MDSCs is increased in oral cavity squamous cell carcinoma (OCSCC) and correlates with pathological markers and prognosis (81). The inhibitory molecules PD-L1 and CD155 are highly coexpressed on MDSCs from HNSCC patients and associated with tumor progression and decreased cytotoxic T cell infiltrates (82). MDSCs can be phenotypically subdivided into two groups, polymorphonuclear MDSC (PMN-MDSC) and monocytic MDSC (M-MDSC). Increased M-MDSC infiltrates are associated with tumor burden after boron neutron capture therapy for HNSCC (83). PD-L1 is expressed to a greater degree on M-MDSCs than on PMN-MDSCs (84).

### TANs.

Neutrophils play a crucial role in HNSCC (67). However, the prognostic significance of TANs in HNSCC is poorly understood (85), which may be related to the variability neutrophil phenotypes, including a cytotoxic antitumor “N1” state and an immunosuppressive protumor “N2” state. The diversity and plasticity of neutrophils contribute to variable immune control, though there is much to be learned.

### Tregs and Bregs.

Tregs are present in the systemic circulation and tumors of patients with HNSCC, and are associated with HNSCC outcomes (86–88). A spectrum of Treg phenotypes likely exists in HNSCC. Neuropilin 1 (NRP1) is preferentially expressed on intratumor Tregs in HNSCC, and NRP1^+^ Tregs are more suppressive and associated with worse outcomes (86). TIM3^+^ Tregs inhibit T cell proliferation, while TIM3 antagonism relieves Treg-mediated immunosuppression in HNSCC (89, 90). CTLA-4 and CD39 are coexpressed on the majority of tumor-infiltrating Tregs, with a greater capacity for suppression than circulating Tregs in HNSCC. CTLA-4^+^ Tregs can suppress cetuximab-mediated ADCC, while their depletion restores NK cytolytic function (14, 91). In addition to Tregs, Bregs with potent immunosuppressive function were identified in HNSCC (discussed below) (92).

### T cell exhaustion.

Exhausted T cell infiltrates are associated with poor outcomes in HNSCCs (93, 94). PD-1– and CTLA-4–expressing T cells are increased in the systemic circulation of HNSCC patients (95). Two subsets of exhausted T cells, CD8^+^PD1^+^TCF1^+^ progenitor exhausted T cells (Tex^prog^) and CD8^+^PD-1^+^TCF1^–^ terminally exhausted T cells (Tex^term^) have been identified in HNSCCs. Tex^term^ T cells were associated with Treg abundance in TME (96). Furthermore, HPV status correlates with PD-L1 expression and T cell exhaustion in HNSCC (Figure 2). T cells in HPV^+^ HNSCCs express higher levels of exhaustion markers, including PD-1, TIM3, LAG3, and TIGIT, compared with those in HPV^–^ HNSCC (97). PD-1^+^ T cells are associated with a favorable outcome in HPV^+^ HNSCC, perhaps serving as a proxy for activated infiltrating T cells responding to the viral antigens (98, 99). In contrast, HPV^–^ HNSCCs tend to have a higher frequency of dysfunctional PD-1^+^ TILs, correlating with a worse overall prognosis (94).

### T cell senescence.

T cell senescence is another important dysfunctional state with a distinct phenotype and function in chronic infections and cancers (100, 101). Senescent T cells highly express senescence associated β-gal but downregulate the costimulatory molecules CD27 and CD28. Senescent T cells are in a state of cell-cycle arrest, with increased cell cycle–regulatory molecules p16, p21, and p53; however this cell population remains metabolically active, producing high amounts of the proinflammatory cytokines IL-2, TNF, and IFN-γ, as well as the suppressive cytokines IL-10 and TGF-β (101). Senescent T cells have been found among TILs in HNSCCs and other cancers (101–103). HNSCC cell lines can directly induce T cell senescence in vitro (103). Importantly, these tumor-induced senescent T cells exert potent suppressive effects on T cell proliferation and function (103). The phenotypic and functional characteristics of senescent T cells may contribute to diminished ICI responses in HNSCC. Improving our understanding of the mechanisms involved in the induction and regulatory role of senescent T cells in HNSCC may lead to novel immunotherapies.

### Metabolic dysregulation in the TME.

Metabolic dysfunction in cancer impacts both tumor and immune cells in HNSCC (104), with the TME characterized by nutrient depletion (105), hypoxia (55), acidity, and suppressive metabolites (106, 107). Tumors manipulate central carbon metabolism, producing lactate through aerobic glycolysis, which can support suppressive Tregs (104) while blunting T and NK cell immune surveillance (108). Tumors also exploit glutamine and lipid metabolism, creating vulnerabilities that hinder T cell function. Furthermore, dysregulated metabolism of glycerolipids, glycerophospholipids, and sphingolipids and upregulation of cholesterol synthesis in HNSCC affect antitumor immune responses (109–111). Tumors can leverage homeostatic mechanisms by release of immunosuppressive metabolites and ions (e.g., spermidine, K^+^) (112, 113), favoring regulatory immune cells, and suppress antitumor responses (55, 104, 114, 115). Glutaminase and glutamate are enriched in HNSCCs (116) and can be targeted with differential effects on cancer cell and immune function, given the metabolic plasticity of T cells (115). Competition for fatty acids and dysregulated lipid metabolites in the TME diminish CD8^+^ T cell responses (109–111, 117). Knowledge of differential dependencies on metabolites and nutrients in the TME could equip us with tools to impair tumor cell viability while enhancing antitumor immunity and abrogating immunotherapeutic resistance.

## Emerging challenges impacting HNSCC response to immunotherapeutic strategies

Potential mechanisms diminishing immunotherapy responses in HNSCC are discussed above. However, several emerging concepts in tumor immunity are being uncovered, which are critical for enhancing immunotherapy against HNSCC.

### Characterizing novel tumor-infiltrating immune cell populations.

Accumulating studies have uncovered the function of certain immune cell types in HNSCC, but there are still cell types whose functions or identity remain unknown or controversial. For example, several studies have demonstrated the existence of γδ T cells in the HNSCC TME, which are associated with poor survival in patients with HNSCC (118–120). However, others found that higher levels of γδ T cells were correlated with lower clinical stages and better OS in HNSCC patients (121). Thus, the precise role of γδ T cells in HNSCC pathogenesis has yet to be elucidated.

Bregs are another poorly defined cell population in HNSCC. Bregs primarily drive immunosuppression, but their effects on tumor progression depend on their phenotypes. Tumor-infiltrating Bregs with a CD19^+^CD38^+^CD1d^+^IgM^+^CD147^+^ phenotype have been identified and express key regulatory molecules including IL-10, CD25, and IDO, contributing to suppression of antitumor immune responses (122). CD19^+^CD24^hi^CD38^hi^ Bregs preferentially localize in tumor tissue rather than peripheral blood and exhibit higher density in the HNSCC TME relative to CD16^+^ B cells (123). Adenosine-producing Bregs (CD39^+^CD73^+^) suppress effector B cells by inhibiting Bruton’s tyrosine kinase phosphorylation via adenosine (124). Notably, increased CD19^+^IL-10^+^ Bregs in OCSCC were associated with CD4^+^ T cell differentiation into Tregs and worse survival outcomes in patients (125). Increased frequencies of atypical memory (CD27^–^IgM^–^IgD^–^) B cells in OCSCC were associated with lower lymph node metastasis, while CD24^hi^CD38^hi^ Bregs were associated with higher stage and nodal metastases (126). Deeper understanding of their function is necessary to develop potential treatment combinations that could improve HNSCC outcomes.

### Dynamics and plasticity of immune cell subsets.

Tregs exhibit a range of phenotypes and variable associations with outcomes in HNSCC patents (56, 127). Various Treg subsets are found in the TME, including thymic selection–derived Tregs (tTregs), peripherally converted Tregs (pTregs), tr-Tregs (tissue-resident Tregs), and follicular Tregs (Tfr Tregs). The functions of these Treg subsets on antitumor responses remain unclear (128). However, the TME augments Treg phenotype, stability, and plasticity, enabling them to switch phenotypic and functional states. Hypoxia in TME affects Treg function and stability. HIF-1α can repolarize Tregs into Th17 cells by upregulating RORγt while attenuating Treg development (129). Nrp1 is required to maintain the stability and function of tumor-infiltrating Tregs. *Nrp1*^–/–^ Tregs produce IFN-γ, which undermines the function of WT Tregs. Hypoxia in the HNSCC TME may drive IFN-γ–induced Treg fragility through HIF-1α (130). Comprehensive understanding of the immunological mechanisms responsible for the control of Treg functionality, plasticity, and instability in TME represents a challenge for HNSCC immunotherapy.

TAMs and TANs exhibit phenotypic plasticity, which can be detrimental to tumor control (131). TME induces the polarization of macrophages and TAMs predominantly exhibiting an M1 phenotype at early tumor stages, when antigen presentation drives antitumor CD8^+^ T cell and NK cell recruitment (132). In the HNSCC TME, tumor cell–derived cytokines and chemokines including IL-6, IL-10, and CCL2 can drive polarization of TAMs toward the M2 phenotype (133). Increased TANs and neutrophil-to-lymphocyte ratio (NLR) were associated with poor prognosis in patients with HNSCC (134, 135). TANs exhibit phenotypic plasticity regulated by TME factors and can be distinguished by an antitumorigenic N1 phenotype or protumorigenic N2 phenotype. TGF-β stimulates N2 and inhibits N1 polarization, while IFN-β promotes N1 and inhibits N2 polarization in the TME. Migration of neutrophils to tumor-draining lymph nodes in HNSCC shapes antitumor immunity in a stage-dependent manner (136). In N0 (without lymph node metastasis) HNSCC, neutrophils can prime T cells, with neutrophil accumulation in T cell–rich zones associated with improved survival. In contrast, neutrophils become immunosuppressive in patients with lymph node metastases and are associated with a poor prognosis. Further understanding of how TAMs and TANs dynamically regulate antitumor immunity is needed to strategically target these cells for enhancing immunotherapeutic responses in HNSCC.

### Distinct metabolic dysregulation in tumor and immune cells.

While we provided a broad overview of metabolic features of TME that impair antitumor immune responses and immunotherapy effectiveness above, several unknowns persist. For example, glycolysis, pentose phosphate metabolism, tricarboxylic acid cycle, and glutamine metabolism are upregulated in HNSCC (137), but we do not know how specific cells such as TILs use these metabolites and what functions they serve in immune evasion. Moreover, tumors can leverage homeostatic metabolites to dysregulate antitumor immunity through unclear mechanisms (114). Distinct metabolic spatial phenotypes have been identified (138); however, the role of these spatial features in driving metastasis, treatment resistance, and immune evasion in HNSCC is unknown. Dysregulated lipid metabolism is also present in HNSCC (139–141). Preclinical data suggest that inhibiting cholesterol synthesis may enhance immunotherapy responses (141). Therefore, additional work is needed to parse the specific mechanisms, substrates, and enzyme kinetics involved in cell-intrinsic metabolism, immune evasion, and response to immunotherapies in HNSCC.

### HPV and HPV-specific immunity in HNSCC pathogenesis.

While much is known about the pathogenesis, progression, and therapeutic outcomes in HPV^+^ HNSCC (142), the molecular processes regulating HPV-mediated immune evasion and responses to immunotherapy remain under investigation (Figure 2). Although HPV viral antigens represent a tumor-specific biomarker, the distribution of viral antigen expression in tumor cells and molecular controls of viral antigen expression must be defined (143, 144). Furthermore, there are several challenges to targeting viral antigens, including MHC-restricted cytotoxic T cell dysfunction and viral molecular mimicry of human proteins (e.g., of HPV16 E7), which could be due to HPV-mediated disruption of antigen processing and presentation (145, 146) and disruption of chemokine and cytokine expression (147, 148). There are likely numerous other undiscovered mechanisms through which HPV drives immune escape, such as metabolic dysregulation, chronic T cell stimulation, impaired coactivation, or genomic alterations. Identifying these mechanisms will equip us to strategically target HPV-intrinsic mechanisms for HNSCC treatment (149).

## Novel therapeutic strategies and combinations

**Figure 3:**
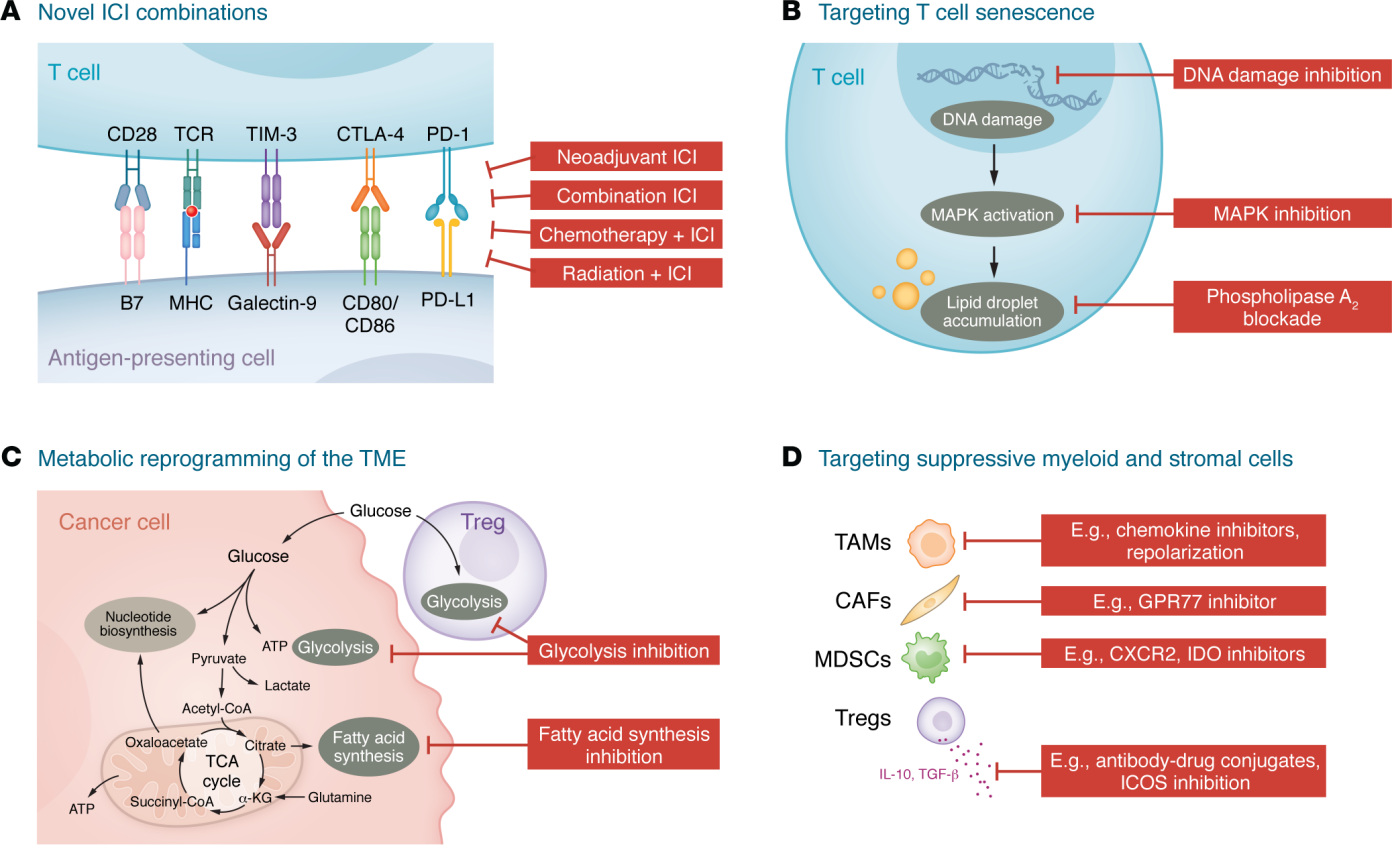
Novel strategies for overcoming immunotherapy resistance in HNSCC. (**A**) ICI can be enhanced by decreasing inhibitory T cell receptor signaling, reinvigorating dysfunctional T cells, and modulating metabolic pathways in cancer cells and suppressive immune populations. Neoadjuvant ICI can increase the antitumor immune response and debulk tumors prior to ablative surgery or cytotoxic therapy. Combining cytotoxic therapies with immunotherapy may also improve the antitumor immune response via dendritic cell activation and T cell priming, activation of pro-death signaling in tumor cells, and release of DAMPs. (**B**) Reversing T cell senescence may also be accomplished through MAPK pathway inhibition, lipid metabolism modulation, and DNA damage blockade. Inhibiting the ability of cancer cells and Tregs to induce T cell senescence offers a novel opportunity for increasing ICI responses. (**C**) Metabolic reprogramming of tumor cells and Tregs also provides novel strategies for HNSCC treatment. (**D**) Targeting suppressive immune and stromal populations will be critical for altering the overall balance of cytotoxic/effector to regulatory responses in the TME.

Despite the aforementioned challenges in enhancing antitumor immune responses to immunotherapy, forthcoming therapeutic options target specific cell populations, patient- and TME-specific immunotherapy combinations, and metabolic reprogramming (Figure 3).

### Neoadjuvant immunotherapy.

Neoadjuvant immunotherapy is poised to revolutionize HNSCC management in the definitive and R/M treatment settings, with the groundbreaking phase III KEYNOTE-689 trial setting the stage for the next era of HNSCC therapy. Multiple neoadjuvant immunotherapy studies have been completed or are ongoing (150–153) (Table 5). Neoadjuvant ICI may enhance long-term tumor control by (i) rejuvenating tumor-specific cytotoxic lymphocytes and trafficking to micrometastatic deposits while enhancing DC presentation of tumor antigens to T cells (154); (ii) increasing antigen-specific responses to a diverse neoantigen repertoire (52); and (iii) increasing systemic immunity (155). Patients in KEYNOTE-689 (NCT03765918) were randomized to receive either neoadjuvant pembrolizumab followed by surgery and adjuvant pembrolizumab or standard surgical resection with adjuvant SoC therapy (156). Unpublished findings prelude improvements in key primary and secondary outcomes (19). Other innovative approaches such as neoadjuvant bintrafusp alfa, an engineered fusion protein targeting PD-L1 and TGF-β signaling, demonstrate enhanced systemic immunity and antigen-specific T cell responses (155). Neoadjuvant α–PD-1/α–CTLA4 therapy using HNSCC samples from the IMCISION trial identified a decrease in activated Tregs and dysfunctional CD8^+^ T cells (157). Leveraging samples from a phase II trial of neoadjuvant nivolumab or nivolumab/ipilimumab in patients with untreated oral OCSCC illustrated increased local and systemic antitumor immunity (151, 158). Neoadjuvant immunotherapy (159), neoadjuvant chemoimmunotherapy (160, 161), and neoadjuvant radiation coupled with immunotherapy (NCT03635164, NCT03247712) (162, 163) in combination are under investigation. Further studies are urgently needed to dissect the mechanisms and optimize treatment combinations driving responses to neoadjuvant immunotherapy in HNSCC.

### Immunotherapy combinations.

Strategically targeting multiple limbs of the immune response may enhance outcomes. Trials testing inhibition of immune-regulatory signaling molecules (e.g., LAG3, TIGIT, TIM-3, CD266, PVRIG, STING, and CD96) are currently underway (Figure 3). These approaches are intended to overcome compensatory upregulation of known and unknown immune-regulatory checkpoint molecules, which may drive adaptive resistance to ICI (164). In one clinical case report, a patient with SoC-refractory HNSCC was successfully treated with the combination of nivolumab and ipilimumab (165). Clinical studies of eftilagimod and relatlimab (LAG-3 inhibitors) for HNSCC are underway. The TACTI-002 trial (NCT03625323) combining eftilagimod with pembrolizumab in second-line metastatic HNSCC observed encouraging antitumor activity (166). Two clinical studies (NCT04080804 and NCT04326257) are treating HNSCC patients with the combination of relatlimab and nivolumab, or nivolumab and ipilimumab, to assess clinical activity. Combined TIGIT and PD-1/PD-L1 blockade is also under investigation, including the combination of neoadjuvant tiragolumab and atezolizumab (NCT03708224 and NCT04665843); α-TIGIT humanized mAbs MK-7684 (NCT05007106) and ASP8374 (NCT03260322) in combination with pembrolizumab; and BMS-986207 in combination with nivolumab (NCT02913313). Additional novel immunotherapy strategies have targeted TGF-β or combined ICI with histone deacetylase inhibition (167, 168) (Table 4). A deeper understanding of T cell activation signaling should drive the rational implementation of forthcoming trials to test for evidence of optimized combinations in HNSCC immunotherapy.

### Targeting senescent T cells in the TME.

Targeting T cell senescence is an emerging concept in tumor immunotherapy (Figure 3) (100, 101, 169). Tregs and tumor cells can induce T cell senescence by triggering effector T cell DNA damage (169–173). Blockage of DNA damage in T cells can prevent T cell senescence and enhance antitumor immunity in both melanoma and breast cancer tumor models (172). Importantly, combining α–PD-L1 checkpoint blockade with DNA damage inhibition to abrogate T cell senescence can synergistically enhance antitumor immunity in those models (172). In addition, activation of MAPK signaling is another important molecular process responsible for development of T cell senescence induced by Tregs and tumor cells in the TME (170–172). Blocking MAPK signaling can also prevent T cell senescence and promote the antitumor efficacy of α–PD-L1 therapy in melanoma and breast cancer tumor models (172). These studies indicate that prevention of senescence in T cells could be an important checkpoint and effective strategy for enhancing HNSCC immunotherapy.

### Targeting suppressive myeloid cell and stromal cell populations.

Immunosuppressive myeloid cell populations can promote tumorigenesis and contribute to the therapy resistance in HNSCC. SX-682, an inhibitor of the myeloid chemokine receptors CXCR1 and CXCR2, inhibited MDSC trafficking and accumulation but enhanced NK cell infiltration, activation, and function in a mouse HNSCC model (174). Immunosuppressive neutrophils upregulate CD36 and fatty acid transport protein 2 (FATP2), which are involved in lipid trafficking in the tumor-bearing mice and human HNSCC. Targeting neutrophil lipid metabolism through FATP2 inhibition reduces the suppressive activity of neutrophils in preclinical models (175). Targeting of the CD47/SIRPa axis on TAMs is another novel opportunity to promote antitumor immunity (176). This is currently being assessed in phase II clinical trials in HNSCC (NCT04854499 and NCT04675294) and oropharynx cancer (NCT05787639). Strategies targeting CAFs in HNSCC are also developing, including CAF reversion or normalization, CAF depletion, targeting ECM, and blocking the immunomodulatory effect of secreted molecules from CAFs or relevant downstream pathways (177). Collectively, these suppressive myeloid and stromal cell–mediated therapeutic responses and should be considered in personalized treatment of HNSCC.

### Leveraging metabolism to reprogram the TME.

A major challenge in treating HNSCC is overcoming metabolic changes in the TME that promote cancer cell–intrinsic treatment resistance and impair antitumor immunity (178, 179). The increased glucose uptake and enhanced glycolysis characteristic of HNSCC appear to make HNSCC susceptible to targeted therapies involving glycolytic inhibitors (179). Key molecules in glycolysis such as HK, PKM2, and GLUT are promising targets for HNSCC treatment. The HK2 inhibitor 2-DG decreases glycolysis and inhibits cell proliferation in HNSCC cell lines (180). Upregulated mTOR signaling is associated with metabolic dysregulation and increased expression of PKM2, PDK1, HIF-1α, LDH, and GLUT1 in HNSCC (181). Inhibition of mTOR signaling with rapamycin reduces tumor growth in HNSCC (182). An oral antihyperglycemic agent, metformin, can affect cell proliferation and antitumor activity in HNSCC through AMPK activation and mTOR inhibition by targeting mitochondrial complex I in HNSCC cells (183). Altering tumor cell metabolism in combination with immune modulation may enhance ICI in HNSCC. A phase II therapeutic trial (NCT04114136) of metformin or rosiglitazone combined with α–PD-1 therapy in solid tumors is currently recruiting participants, aiming to determine whether these compounds synergize with α–PD-1 therapy. Notably, more-selective strategies specifically targeting metabolism in tumor cells will need to avoid inadvertent impairment of tumor-specific cytolytic T cell function. Furthermore, effective metabolic interventions can synergize with immunotherapy and offer novel and promising strategies for enhancing the effectiveness of ICI.

Lipid metabolism reprogramming is also a critical hallmark of HNSCC linked to the carcinogenesis and development of HNSCC (109). Fatty acid synthase (FASN) is overexpressed and associated with aggressiveness, prognosis, and risk of metastasis in OCSCC. Inhibitors targeting FASN, including TVB-3166, C75, and triclosan, have anticancer effects on OCSCC cell lines, with decreased proliferation, migration, and invasion (184, 185). The FASN inhibitor orlistat reduces the volume of primary tumors and lymph node metastases in an orthotopic OCSCC mouse model (186). Notably, metabolic differences between HPV-related and carcinogen-driven HNSCCs should be taken into account to identify the optimal metabolic treatment strategy, although targeting energetic metabolism is a promising anticancer therapy for HNSCC treatment (187).

## Future perspectives

Immunotherapy is a promising strategy in HNSCC. However, several hurdles remain. Potential mechanisms of ICI resistance include molecular and immune heterogeneity coupled with high levels of regulatory immune cell populations, T cell dysfunction, and metabolic dysregulation in the TME. To enhance ICI, more effort is needed to define the function and role of novel tumor-infiltrating immune cell populations, the tumor-intrinsic adaptations that promote immune suppression, the developmental trajectories and plasticity of immune cell populations, and strategies for overcoming metabolic dysregulation. Overcoming challenges to ICI by targeting novel combinations of immune checkpoint molecules and reinvigorating dysfunctional T cells will also be important. Leveraging novel techniques, including single-cell RNA sequencing, spatial multispectral imaging, and multiomics strategies to better understand the TME at a single-cell and molecular resolution will aid these endeavors.

Identification of reliable biomarkers for predicting immunotherapy response in HNSCC remains a critical area of research. PD-L1 expression has been the most widely studied biomarker and is routinely used to guide the use of ICIs. However, its utility is limited due to variable expression thresholds and response rates. Tumor mutational burden (TMB) and microsatellite instability (MSI) are additional biomarkers that have shown promise in other cancer types, but their predictive value in HNSCC has been less robust (188, 189). Emerging biomarkers, such as circulating tumor DNA (ctDNA), and the composition of the TME, including density and activity of infiltrating T cells and regulatory cells, are being investigated. Additionally, expression of HPV-related viral antigens may guide immunotherapies such as tumor vaccines or engineered TCR T cells (50). Advances in multiomics profiling and artificial intelligence are facilitating the discovery of composite biomarkers that integrate genetic, transcriptomic, and proteomic data, which may enhance the precision of immunotherapy in HNSCC.

Developing novel combination therapies targeting regulatory cell subsets, dysfunctional T cells, and the dysregulated metabolite and nutrient milieu of the TME will also be important for advancing immune responses and long-lasting systemic immunity to HNSCC. Reversing T cell exhaustion secondary to chronic antigen stimulation will require a detailed understanding of how the frequency of antigen-positive tumor cells and strength of the peptide/MHC-TCR interaction influence T cell fate (190). Developing computational and bench models to identify and quantify putative neoantigens and test their binding affinity may help us understand which T cell infiltrates require reinvigoration via immune checkpoint antagonism versus those that would benefit from targeting regulatory cell subsets. Furthermore, overcoming the nutrient-depleted, acidic, hypoxic TME will further optimize responses to these therapies. In addition, dissecting the fundamental immune microenvironment differences and heterogeneity between HPV^+^ and HPV^–^ HNSCC will elucidate the mechanisms by which tumors evade the endogenous immune response. In parallel, it will be important to define how other viruses such as Epstein-Barr virus influence the tumor immune landscape in HNSCC. Finally, targeting metabolic vulnerabilities such as lactate, glutamine, polyamine, and lipid metabolism is urgently needed in HNSCC (114, 115, 171). Mounting data highlight the plasticity and adaptability of immune cells — such as Tregs’ use of lactate as a fuel source and alternative T cell nutrient utilization — while tumor cells succumb to metabolic disruption (115). Identifying how metabolites and nutrients are differentially used by tumors and immune cells will permit strategic development of novel therapies.

## Figures and Tables

**Table 1:**
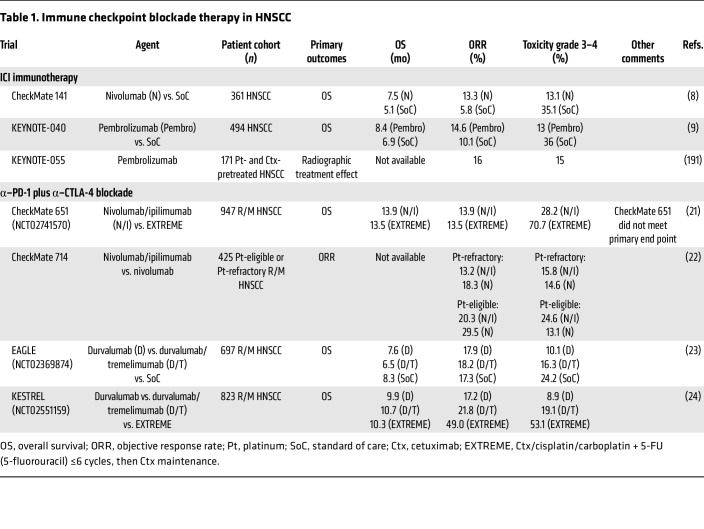
Immune checkpoint blockade therapy in HNSCC

**Table 2:**
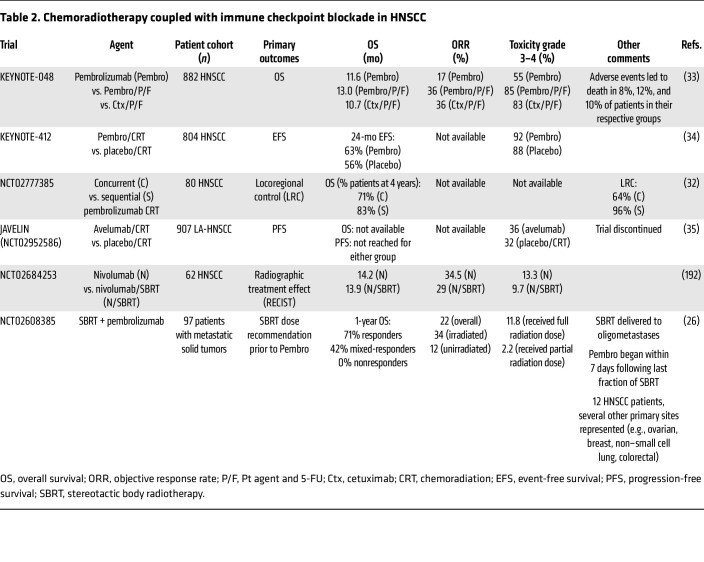
Chemoradiotherapy coupled with immune checkpoint blockade in HNSCC

**Table 3:**
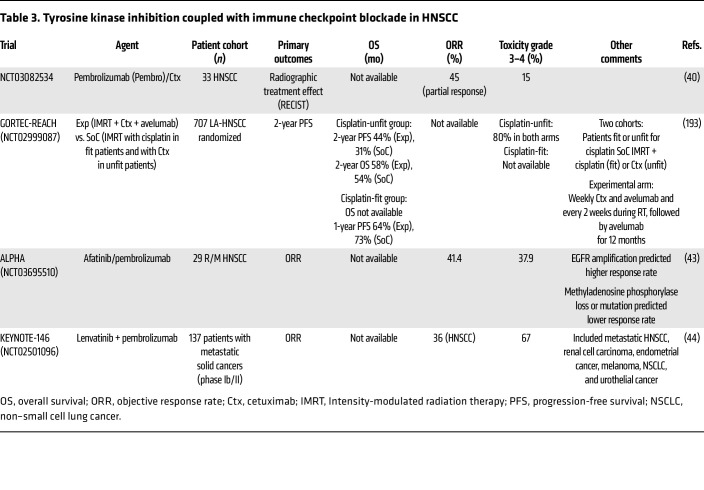
Tyrosine kinase inhibition coupled with immune checkpoint blockade in HNSCC

**Table 4:**
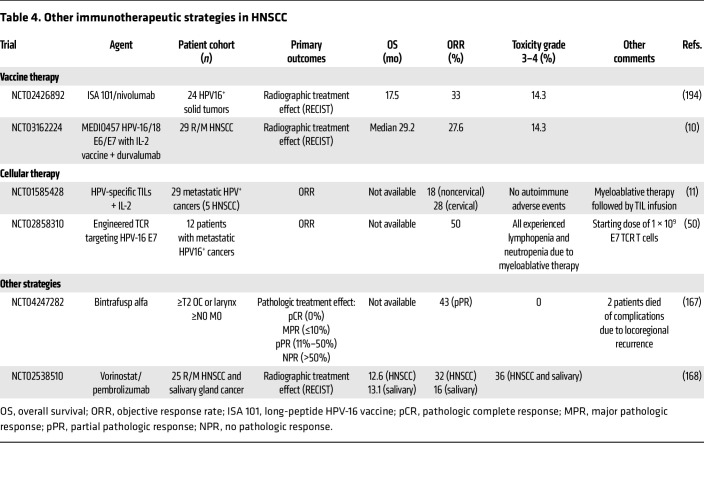
Other immunotherapeutic strategies in HNSCC

**Table 5:**
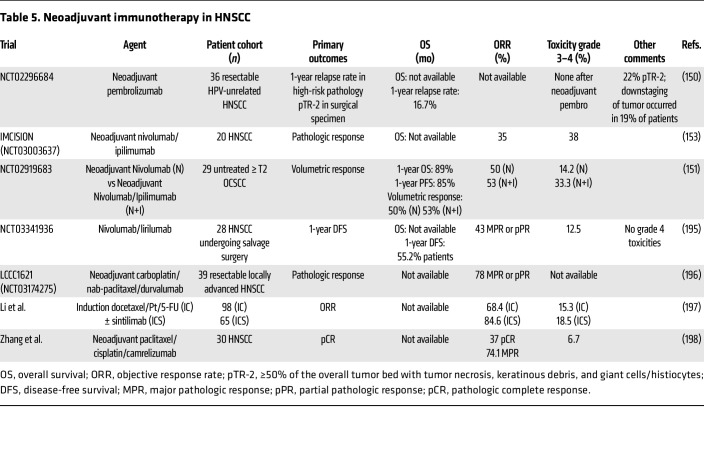
Neoadjuvant immunotherapy in HNSCC

## Version 1 04/15/2025

Electronic publication
